# Analysis of volatile fraction of sweetie (*Citrus maxima *×* Citrus paradisi*) and its parent fruit using proton transfer reaction mass spectrometry

**DOI:** 10.1007/s00706-018-2229-4

**Published:** 2018-08-09

**Authors:** Anna Różańska, Dorota Sieńska, Tomasz Dymerski, Jacek Namieśnik

**Affiliations:** Department of Analytical Chemistry, Faculty of Chemistry, Gdansk University of Technology, Narutowicza 11/12, 80-233 Gdańsk, Poland

**Keywords:** Fragrances, Mass spectroscopy, Natural products, Proton transfer reaction, Principal component analysis

## Abstract

**Abstract:**

The quality of the fruit is affected by several main ingredients and the aroma plays a fundamental role during the selection of fruit by consumers. In the case where several fruit have similar aromas and only one of them has specific health properties, it is very important to find the differences in the volatile organic compounds (VOCs) composition to distinguish these samples. Such situations are often found for hybrid fruit. Sweetie is a hybrid of grapefruit and pummelo. Sweetie fruit is characterized by high antioxidant potential and a positive effect on human health. The aim of this study was to verify the unique volatile compositional traits of three species of citrus fruit. Proton transfer reaction Time-of-Flight mass spectrometry (PTR-TOF-MS) was utilized to obtain the mass-resolved fingerprints of VOCs. The chemical formula of these VOC masses was tentatively identified. Principal component analysis was performed to evaluate the differences between the groups.

**Graphical abstract:**

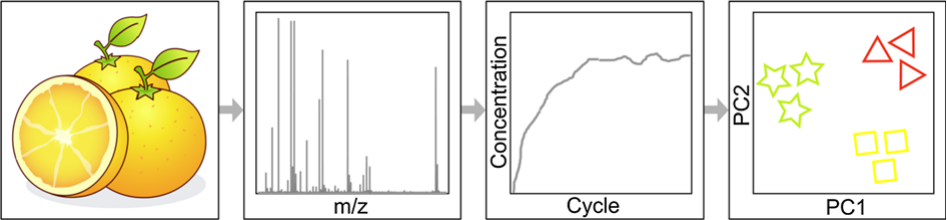

## Introduction

Citrus fruit are one of the most commonly consumed fruit varieties in the world. Experts at the Organization for Economic Co-operation and Development estimate that in 2017 over 90 million tons of citrus fruit were produced [1].

In addition to very good taste, these fruits are also characterized by pro-health properties. This is due to the high content of antioxidant compounds. Citrus fruit have a high content of ascorbic acid, phenolic compounds, and flavonoids [2–4]. Moreover, these kind of fruit is a good source of many vitamins and minerals necessary for the proper functioning of the human body [5]. Furthermore, it has been found that many of the chemical substances, determined in citrus fruit, have antioxidant properties, which can promote the elimination of cancer cells [6]. Gorinstein et al. proved a protective effect of high citrus fruit in the risk of stomach cancer [7]. A high intake of these fruits may reduce the risk of degenerative diseases [8].

In recent years, sweetie fruit (*Citrus maxima *×* Citrus paradisi*) has become more and more popular. It is a hybrid between white grapefruit (‘Marsh’ *Citrus paradisi Macfadyen*) and pummelo (*Citrus grandis ‘*Osbeck’) invented in 1962 [9]. This fruit is also known as ‘Oroblanco’ grapefruit. The taste of the sweetie resembles the taste of grapefruit, but it is much sweeter, because it contains almost twofold higher content of sugars compared to ‘Marsh’ grapefruit [10]. Phenol content and the antioxidant potential are also significantly higher in sweetie than in grapefruit [11, 12]. Based on research of juice squeezed from sweetie fruit, it was found that this juice contains large amounts of bioactive compounds and has a high antioxidant potential [13]. And including it in the daily human diet can positively affect the levels of serum lipids, albumin, and fibrinogen [14]. The higher antioxidant capacity and sweet flavour of sweetie could make these new kinds of citrus fruit preferable in human diets.

Fruit aroma is an extremely important factor influencing the attractiveness of a given product to the consumer. Based on the aromas of food products (including fruit), their taste and quality can be pre-determined. The chemical aroma of citrus fruit is mainly evaluated by gas chromatography (GC) techniques. The research is carried out mainly on samples of fruit peels or extracted essential oils [15–17]. The pulp and juices squeezed out of these fruits are analyzed less frequently [10, 18–20]. The application of gas chromatography technique is time-consuming and labour-intensive. Moreover, GC is characterized by a relatively long time of analysis; therefore, alternative methods for the analysis of volatile compounds are sought.

Another advanced analytical technique which might be used to determine VOCs in the fruit samples is a proton transfer reaction mass spectrometry (PTR-MS). The PTR-MS technique was invented in 1998 by Lindinger et al. [21]. PTR-MS system allows for real-time online VOCs monitoring at ultra-low concentration as a few ppt_v_ in real-time. Moreover, when the PTR-MS is equipped with a Time-of-Flight (TOF) analyzer, the application of this technique provides to obtain mass resolutions greater than 6000 [22]. PTR-MS is a modern technique generally used to measure VOCs fingerprints. Compared to traditional gas chromatography mass spectrometry (GC–MS), PTR-MS enable to analyze the entire VOCs profile of samples without a sample preparation step, with high sensitivity and low detection limits. This technique is, therefore, widely used in many areas of food analysis [23–25], including fruit analysis, especially for determining the stage of ripening [26–29].

In the case of the hybrid fruit, namely sweetie and its parent fruit can be noted that pulps and juices from these fruits are visually almost identical. The aromas of these citrus fruit are also very similar. However, sweetie fruit is characterized by better health-promoting properties. For this reason, it is very important to find differences in the aromas of these fruits which allow distinguishing them. Nevertheless, there are no literature reports about authenticity indicators of above-mentioned types of citrus fruit pulps, including also the use of proton transfer reaction mass spectrometry. Therefore, the aim of this study was to compare sweetie, grapefruit and pummelo pulps regarding to their aroma. For this reason, the advanced analytical method was elaborated with the use of PTR-MS in connection with chemometric method—principal component analysis (PCA). The application of PCA allows to present the differences in VOCs composition between hybrid—sweetie and their parent fruit.

## Results and discussion

The pulp of three species of citrus fruit: grapefruit, pummelo, and a hybrid of those two—sweetie were subjected to PTR-MS analysis. Mass spectra were generated for six replicates of each fruit type samples. The mass spectral data were used as fingerprints. The masses present in each sample and their corresponding signal intensities (cps) served as a pattern for sample comparison.

**Fig. 1:**
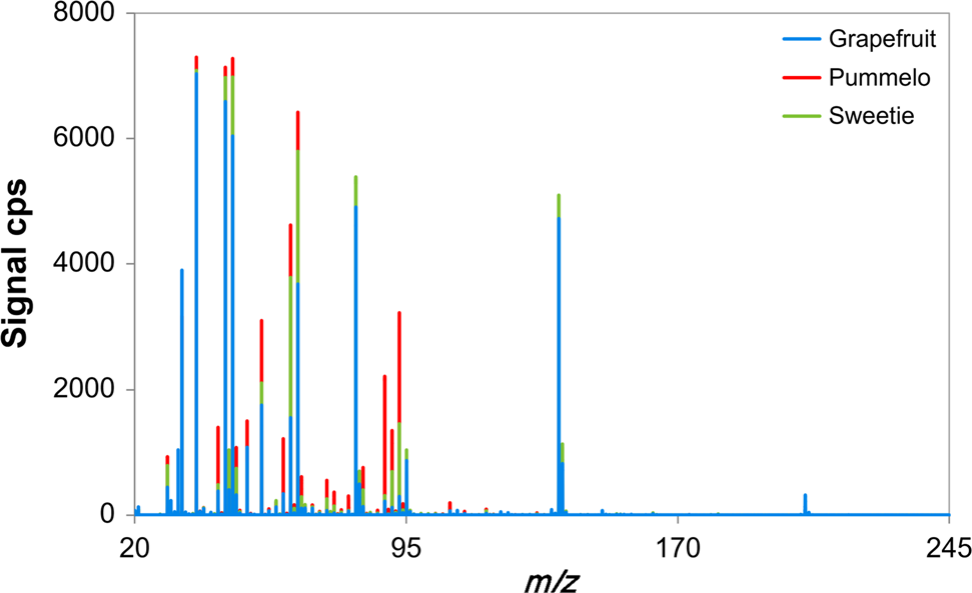
Mean fingerprint mass spectra of the headspace of samples of the three species of citrus fruit: grapefruit, pummelo, and sweetie

Mean mass spectra for three types of citrus fruit samples are displayed in Fig. 1. The five most abundant ions (descending order) for grapefruit were *m/z* = 137, 81, 47, 45, 37; for pummelo *m/z* = 63, 65, 47, 45, 37; and for sweetie *m/z* = 81, 65, 47, 45, 37. There were almost twenty common major ions with quantitative differences. The mass fingerprints of the three types of citrus fruit showed hardly any qualitative differences, the same ions were observed for the three species of fruit, but concentrations varied. Due to the very small differences in the aroma of the studied fruit, it is very difficult to distinguish them from each other. For this reason, in the presented research the PTR-MS technique with the chemometric methods were combined.

Application of the PTR-MS technique allowed to pre-identify chemical compounds from the obtained mass spectra. However, protonated molecules may fragment, for this reason, relatively complex spectra can arise which are not easy to interpret. Therefore, it is very important to conduct the identification based on the fragmentation pattern of the studied chemical compound [30–32]. It should be remembered that this is a tentative identification, which consists in the assignment of the detected ions to the fragmentation patterns characteristic of given chemical compounds, as well as on a comparison with literature data.

For the tentative identification of chemical compounds which were detected in the volatile fractions of the grapefruit, pummelo, and sweetie samples, the crucial ions for distinguishing the studied fruit were selected. To determine which of the analytes present in the headspace of the samples had the greatest impact on the result of the statistical analysis; an analysis of variance ANOVA was carried out. As a result of this operation, the 10 ions shown in Table 1 were selected.

**Table 1:** Tentative identification and determination of VOCs done by PTR-TOF-MS for grapefruit, pummelo, and sweetie samples

No.	*m/z*		Formula	Mean concentration ± SD/ppm_v_			Tentative identification	CAS no.	References
	Measured	Theoretical		Grapefruit	Pummelo	Sweetie			
1	45.0358	45.0334	C_2_H_5_O^+^	3.61 ± 0.36	7.94 ± 0.24	5.72 ± 0.11	Acetaldehyde	75-07-0	[27, 29, 33]
2	61.0269	61.0284	C_2_H_5_O_2_^+^	0.309 ± 0.019	1.73 ± 0.12	< LOQ	Acetic acid	64-19-7	[29]
3	67.0564	65.0543	C_5_H_7_^+^	0.1495 ± 0.0084	0.2234 ± 0.0041	0.1247 ± 0.0035	Fragment of terpenes	–	[34]
4	69.0676	69.0698	C_5_H_9_^+^	< LOQ	0.1513 ± 0.0086	< LOQ	Isoprene	78-79-5	[27]
5	75.0779	75.0441	C_3_H_7_O_2_^+^	< LOQ	0.223 ± 0.013	< LOQ	Methyl acetate	79-20-9	[29, 31]
6	81.0727	81.0698	C_6_H_9_^+^	1.39 ± 0.11	0.154 ± 0.011	2.61 ± 0.12	Fragment of terpenes	–	[27, 33, 34]
7	83.0751	83.0855	C_6_H_11_^+^	< LOQ	0.205 ± 0.020	0.1405 ± 0.0078	2-Hexenol/3-Hexenol	928-95-0	[27, 31, 33]
8	89.0568	89.0597	C_4_H_9_O_2_^+^	< LOQ	1.53 ± 0.14	< LOQ	Ethyl acetate	141-78-6	[29, 31, 33]
9	93.0901	93.0698	C_7_H_9_^+^	< LOQ	1.61 ± 0.16	0.374 ± 0.031	Fragment of monoterpenes	–	[27, 33, 34]
10	95.0821	95.0855	C_7_H_11_^+^	0.276 ± 0.017	< LOQ	0.622 ± 0.033	Fragment of monoterpenes	–	[33, 34]

*LOQ* limit of quantitation, *SD* standard deviation, mean ± SD of 6 measurements

In Table 1 the concentrations quantified in the headspace of grapefruit, pummelo, and sweetie samples were also presented. As it can be seen in several cases (e.g., *m/z* = 61, 69 or 75), the concentrations of detected chemical compounds were lower than the limits of their quantification. In the case of other chemical compounds, differences in their content in the fruit volatile fractions could be small (e.g., *m/z* = 67) or differ significantly (e.g., *m/z* = 93). For *m/z* = 67, the differences in concentrations between three citrus fruit were amounted to less than 100 ppb_v_. However, in the case of *m/z* = 93, the concentrations differences in the volatile fractions of the grapefruit and pummelo were up to an order of magnitude (1 ppm_v_).

Ions common to all three varieties of studied citrus fruit were *m/z* = 45, 67, 81 and 93. Farneti et al. detected ion *m/z* = 45 in samples of blueberry in different ripening stages. During research it was found that this ion can be identified as acetaldehyde [33]. The three remaining ions are characteristic of terpenes. Tani et al. used PTR-MS to investigate the fragmentation patterns of compounds in the monoterpene family. Based on these results, the ion *m/z* = 67 can originate from β-pinene or limonene, the *m/z* = 81 from the linalool and the *m/z* = 93 from the *p*-cymene [34]. Ions detected only in grapefruit and pummelo samples were *m/z* = 61 and 89. Bianchi et al. studied the composition of the headspace of various peach varieties using PTR-MS. During research, ion *m/z* = 61 was determined and it was found that it is characteristic for the acetates or acetic acid [29]. According to studies carried out by Buhr et al. ion *m/z* = 89 may be associated with the presence of acetates, mainly ethyl acetate [31]. On this basis, it can be concluded that acetates can affect the aroma of parent fruit, but are not specific to their hybrid. Ion *m/z* = 83 was present in both of the studied volatile fractions: in the parent fruit (pummelo) and in the hybrid (sweetie). This ion was identified during the study of the volatile fraction of mango fruit by Taiti et al. Its detection can be determined by fragments of C6 compounds, such as hexenals or hexenols [27]. A similar situation was with the *m/z* = 95 ion, which was detected in the aromas of both sweetie and grapefruit, and it is characteristic for terpenes, e.g., limonene or 3-carene [34]. In addition, the differentiating ions to the studied fruit were ions *m/z* = 9 and 75. They were detected only in pummelo samples. These ions (*m/z* = 69 and 75) can originate from isoprene, the terpenes fragment [27], and from methyl acetate [31], respectively.

**Fig. 2:**
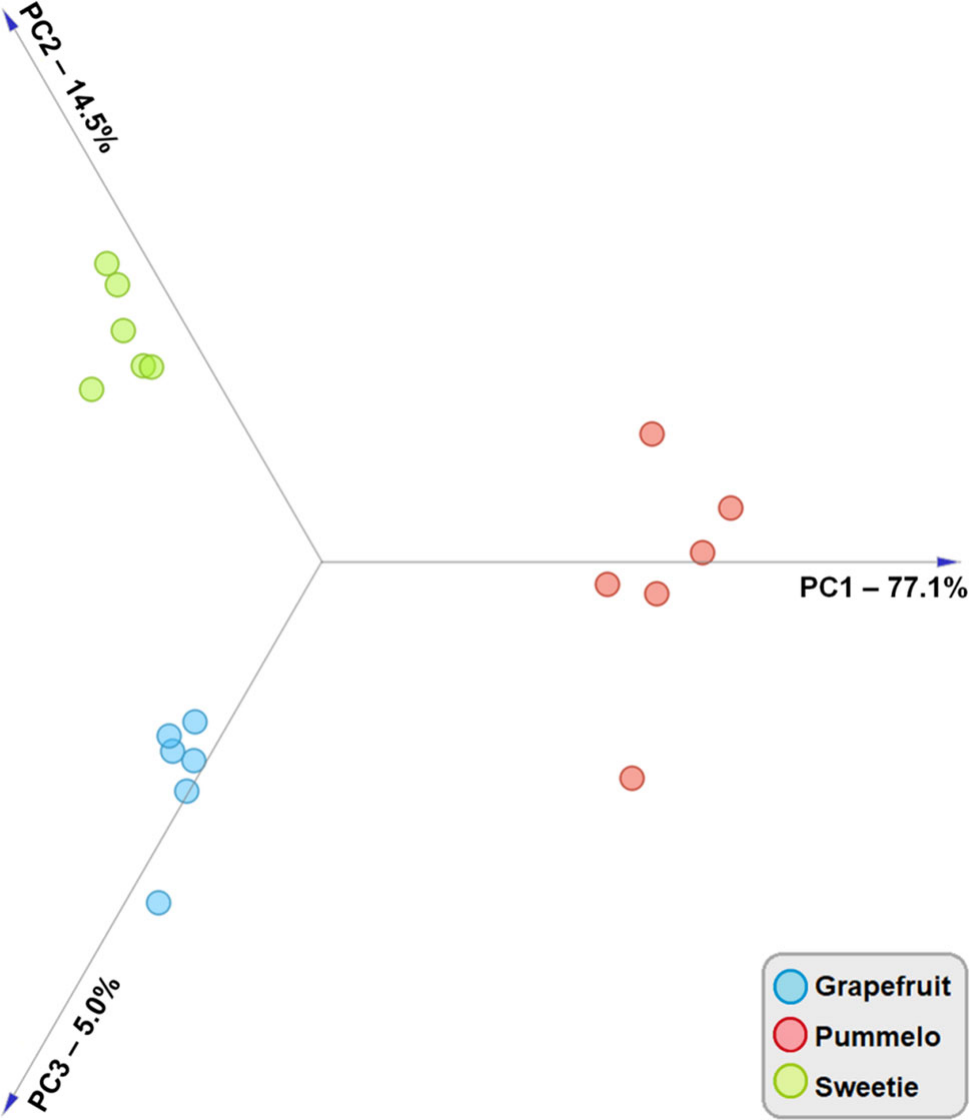
Linear projection of three principal components obtained for samples of grapefruit, pummelo, and sweetie

Pre-identified chemical compounds with concentrations were then used as input data for chemometric analysis. A PCA was calculated based on the concentrations of the 10 volatiles listed in Table 1, which were quantified in the aroma of the three citrus species to further explore the differences among their pulp samples. The result of PCA is shown in the graph (Fig. 2).

Based on this result (Fig. 2), it was found that use the PTR-TOF-MS device makes it possible to distinguish the studied citrus fruit. The first three principal components (PCs) of the model explained 96.6% of the total variance among the samples with contributions of 77.1% by PC1, 14.5% by PC2, and 5.0% by PC3, respectively. Due to the comparatively high variance explained by the model, PCA permitted a clear-cut separation of samples into three clusters, according to the botanical origin of the samples.

**Fig. 3:**
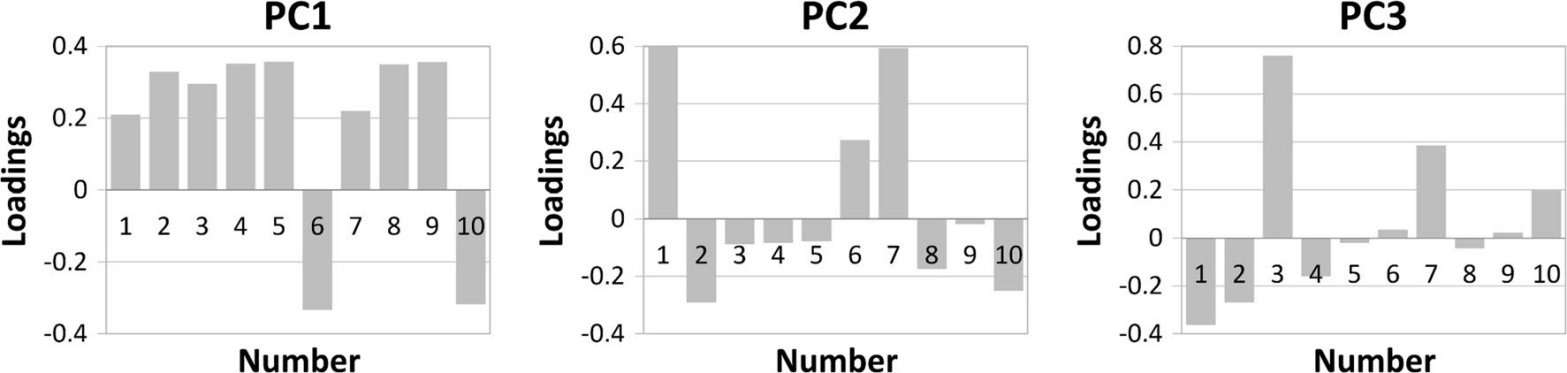
Loadings plots for three principal components obtained for samples of grapefruit, pummelo, and sweetie (numbers correspond to Table 1)

Examples of the three PCs loadings plots are given in Fig. 3. It should be noted that the first principal component was determined by all 10 selected chemical compounds listed in Table 1. On the loadings graph for the PC1 were exhibited 8 high positive loadings (compounds from 1 to 5 and from 7 to 9) and 2 high negative loadings (compounds 6 and 10). The separation along the second principal component mainly was depended on the concentration of compound 1 (acetaldehyde) and compound 7 (2-hexenol or 3-hexenol). Both of these compounds were high positive loadings for the PC2. Furthermore, the distinction along the third principal component was depended mainly on the high positive loading compound 3—ion *m/z* = 67, which was characteristic for terpenes and was tentatively identified as β-pinene or limonene. In addition, compound 7 and compound 1 concentrations (positive and negative loading, respectively) also influence the differentiation of the samples along PC3.

**Fig. 4:**
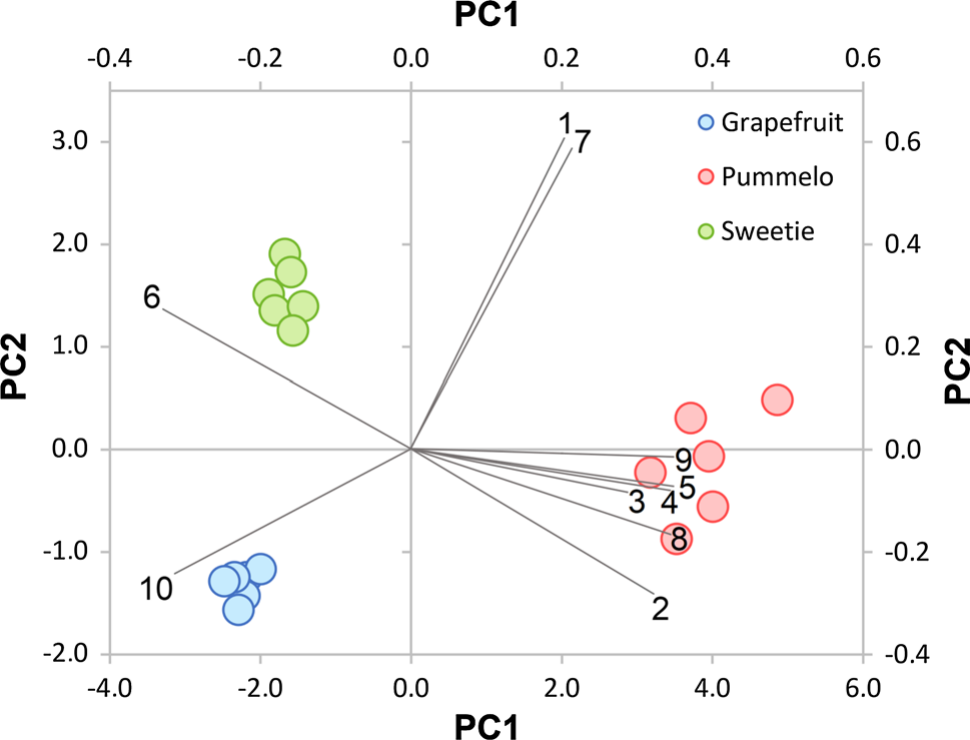
PCA biplot of volatile compounds of grapefruit (blue), pummelo (red), and sweetie (green) pulp. Variables explained: (1) acetaldehyde; (2) acetic acid; (3) β-pinene; (4) isoprene; (5) methyl acetate; (6) linalool; (7) 2-hexenol; (8) ethyl acetate; (9) *p*-cymene; (10) 3-carene

In Fig. 4 biplot of grapefruit, pummelo, and sweetie samples with selected volatiles was presented. Two principal components (PCs) were obtained from the volatile concentrations in the citrus fruit pulps. These PC were accounted for almost 92% of the cumulative percentage of total variations. Based on this result (Fig. 4), it can be observed that, the samples of pummelo could be distinguished from samples of other citrus fruit along PC1. However, samples of grapefruit and sweetie were separated along PC2, so the variance between these three groups of objects was mainly obtained by PC2. PCA-biplot allows to correlate between the selected volatile compounds and the groups of objects (citrus fruit samples). For example: acetic acid, β-pinene, isoprene, methyl acetate, ethyl acetate, and *p*-cymene were positively correlated with pummelo. It should be noted that these compounds were responsible for discrimination of pummelo samples from others citrus fruit. In turn, limonene was associated with grapefruit samples and linalool was related to sweetie.

Based on the data included in Table 1 and Fig. 4 it can be observed that terpenes are the major volatiles of citrus fruit. Cheong et al. found that the terpenes constituted almost 72% of the content of the volatile fraction from freshly squeezed pummelo juice [19]. However, Zheng et al. proved that in the case of sweetie and grapefruit pulp, the content of terpenes in the volatile fraction is 89 and 92%, respectively [10]. For this reason, the volatile fraction for pummelo can be distinguished from other samples already along to PC1. In addition, one of the chemical compounds which differentiated pummelo samples from others was acetic acid, because pummelo fruit are slightly acid, unlike white grapefruit or sweetie samples [19]. The samples of grapefruit and sweetie aromas were very similar, but according to the determination of the concentrations of two characteristic chemical compounds, we could distinguish them. The compounds characteristic for grapefruit and sweetie were, respectively, 3-carene and linalool. These terpenes have already been marked in the fruit pulp samples by the research team Zheng et al. using GC–MS. Comparing these data with the results listed in Table 1, similar relationships could be observed (e.g., sweetie contained twofold higher concentration of linalool than grapefruit), which confirms the correct identification of selected ions.

## Conclusion

An occurrence of VOCs in fruit pulp determines their aroma. Aroma is an important quality property of food products, especially for fruit. Flavour of fruit depends mainly on botanical origin but in the case of hybrid fruit, the differences in aroma can be insignificant. Wherefore, the advanced analytical techniques should be used to determine these dissimilarities. In this work, the PTR-TOF-MS to profile the samples of grapefruit, pummelo, and sweetie was employed. The study has demonstrated the different volatile profiles of three varieties of citrus fruit. The described procedure enables the possibility to distinguish citrus fruit due to their botanical origin. The application of the PCA allows to select volatile organic compounds characteristic for a studied citrus species. Based on the obtained results, it might be concluded that esters and acetic acid was associated with pummelo samples and limonene and linalool were related to grapefruit and sweetie samples, respectively. In summary, the proposed methodology allows to distinguish the grapefruit, pummelo, and sweetie samples based on their volatile fraction.

## Experimental

### Plant material

The VOC profiling was performed on ripe citrus fruit belonging to three species: ‘Marsh’ grapefruit (*Citrus paradisi Macfadyen*), pummelo (*Citrus grandis* ‘Osbeck’) and a hybrid of these two fruit, namely sweetie (*Citrus maxima *×* Citrus paradisi*, also known as ‘Oroblanco’). All fruit samples were purchased at local markets in Gdansk. The subject of the research was the pulp of selected fruit. Immediately after the purchase, each fruit was washed and peeled. The fruit pulp was homogenized using an agate mortar. After that, 5.0 ± 0.1 g of the crushed pulp for each fruit was transferred to 20 cm^3^ glass vials. The vials were closed with a silicone-PTFE membrane cap. Six samples were prepared for each fruit. Each repetition was made of another piece of fruit.

### Analysis of VOCs

Fruit from the three citrus fruit varieties were analyzed by a commercial PTR–TOF 1000 Ultra (Ionicon GmbH, Innsbruck, Austria). Before analysis, samples were incubated at 40 °C for 5 min. Fingerprinting of fruit samples was evaluated using H_3_O^+^ as reagent ion for the proton transfer reaction. VOCs were then measured by direct injection of the samples’ headspace into the PTR-TOF-MS drift tube with flow rate of 50.0 cm^3^/min. The operating parameters in the drift tube were set, respectively, at: pressure 2.6 mbar, temperature 70 °C and *E*/*N* value of 120.0 Td which allows to avoid excessive formation of water clusters [35]. Ambient air passed through a carbon filter (Supelpure HC, Supelco, Sigma-Aldrich, Steinheim, Germany) was used as carrier gas. MS data between 20.0 and 245.0 atomic mass units (amu) was collected. Sample measurement was performed in 30 cycles resulting in an analysis time of 30 s/sample. Blank measurements were carried out between samples to monitor background air. Between two measurements 1 min interval was kept to avoid memory effects. After drawing from the drift tube, protonated ions were separated according to their mass-to-charge (*m/z*) ratio [22]. The spectra were recorded with IoniTOF v. 2.4.40 software and the raw data were processed with PTR-MS Viewer v. 3.2.3.0. Compounds were tentatively identified based on the measured protonated masses and reports of previous literature about fragmentation patterns, and isotopic ratios. VOCs concentrations are expressed in ppb_v_ (part per billion by volume) and has been calculated from peak areas according to the formula described by Lindinger et al. [21]. During research, a constant reaction rate coefficient of 2 × 10^−9^ cm^3^/s was used. The limit of quantification for the spectra was set at ten standard deviations of the background noise registered for a blank sample.

### Statistical analysis

To obtain fingerprints, the spectrum of the blank sample was subtracted from the averaged spectra for samples of three citrus species. Data from PTR-MS measurements were also used as input for statistical analyses. For this purpose, Orange v. 3.3.9 software (Bioinformatics Lab, University of Ljubljana, Slovenia) was used. To obtain particular signals, the spectrum of the blank sample was subtracted from the averaged spectra for individual samples. The data were then normalized by centring to the mean and scaling by standard deviation. From the prepared data set, 10 ions—which had the greatest impact on the result of statistical analysis—were selected basis on the analysis of variance ANOVA. Concentrations for 10 selected ions were used as input data for PCA. Unsupervised PCA method made it possible to visualize the distinction between grapefruit, pummelo, and sweetie samples based on their volatile substances.
